# Elucidating the role of mitochondrial Alzheimer’s Disease risk gene LACTB in myeloid cells

**DOI:** 10.1002/alz.086622

**Published:** 2025-01-03

**Authors:** Carmen Romero Molina, Wen Yi See, Tulsi Patel, Yiyuan Liu, Edoardo Marcora, Alison M. Goate

**Affiliations:** ^1^ Icahn School of Medicine at Mount Sinai, New York City, NY USA; ^2^ Ronald M. Loeb Center for Alzheimer’s disease, New York City, NY USA; ^3^ Ronald M. Loeb Center for Alzheimer’s Disease, Friedman Brain Institute, Icahn School of Medicine at Mount Sinai, New York, NY USA; ^4^ Department of Genetics and Genomic Sciences, Icahn School of Medicine at Mount Sinai, New York, NY USA; ^5^ Icahn School of Medicine at Mount Sinai, New York, NY USA; ^6^ Ronald M. Loeb Center for Alzheimer’s disease, New York, NY USA

## Abstract

**Background:**

While compelling evidence highlights the importance of myeloid cells in the etiology of Alzheimer’s Disease (AD), the relevance of immunometabolism still requires further exploration. Our analysis integrating AD genetics and myeloid cell genomics shows that lower levels of LACTB expression in myeloid cells is protective against AD, a finding supported by proteomics studies. As a mitochondrial active‐site serine protein, LACTB has implications for mitochondrial morphology and bioenergetics. LACTB levels are also linked to succinyl‐carnitine levels, a metabolite predicted to be protective for AD risk. Furthermore, LACTB has been associated with tumorigenesis and obesity, suggesting a potential role in cell dynamics and lipid metabolism, although its function remains elusive.

**Method:**

Human immortalized THP‐1 cells monocytes were differentiated into macrophages and treated with small‐interfering RNA to knock‐down LACTB (KD) expression. CRISPR‐edited human induced pluripotent stem cells (hiPSC) were generated to knock‐out (KO) LACTB and differentiated into microglia cells (iMGL). Metabolomic and lipidomic analyses were outsourced with collected samples, and functional assays evaluating how LACTB alters myeloid cell functions were performed.

**Result:**

Our studies revealed an upregulation of LACTB expression upon differentiation (in iMGLs compared to iPSCs, and in THP‐1 macrophages compared to monocytes) and LPS stimulation. Downregulation of LACTB in THP‐1s and iMGLs resulted in elevated succinyl‐carnitine levels, and THP‐1 LACTB KD cells showed an increase in histone succinylation levels, which might indicate epigenetic modulation. We also detected a reduction in nascent protein synthesis levels in our LACTB KD and KO models. Additionally, lipidomics showed a decrease in acylglycerides, and an increase in cholesterol efflux was observed in THP‐1 LACTB KD cells.

**Conclusion:**

LACTB potentially modulates myeloid cell function by modifying protein synthesis and lipid metabolism. Future directions include a novel xenotransplantation model involving the direct introduction of human microglia precursor cells into the mouse brain to study the effects of LACTB on microglia in‐vivo and in the context of disease using 5xFAD mice. The distinct identity of LACTB as an enzyme, coupled with its protective phenotype upon reduced expression and the potential of succinyl carnitine as a biomarker, makes it a promising therapeutic target in AD.

